# Reasons for non-compliance among patients with hypertension at Vanga Hospital, Bandundu Province, Democratic Republic of Congo: A qualitative study

**DOI:** 10.4102/phcfm.v1i1.68

**Published:** 2009-08-18

**Authors:** Jean-Pierre Fina Lubaki, Langalibalele Mabuza, Nomsa Malete, Patrick Maduna, John V. Ndimande

**Affiliations:** 1 Vanga Hospital, Democratic Republic of Congo; 2 Department of Family Medicine and Primary Health Care, University of Limpopo, South Africa; 3 Head Office, Gauteng Department of Health, South Africa

**Keywords:** hypertension, non-compliance, side effects, antihypertensive drugs, poor knowledge on hypertension

## Abstract

**Background:**

Hypertension is a serious public health challenge in both economically developing and developed countries. Patients on outpatient medication for hypertension at Vanga Hospital in the Democratic Republic of Congo (DRC) often present with uncontrolled hypertension and some with hypertension emergencies. On enquiry, the problem appeared to revolve around compliance.

**Method:**

The study was a qualitative, descriptive study using the focus group interview technique for data collection. Subjects were purposely selected. Interviews were conducted from 23 March to 19 July 2006. Three focus groups were formed: The first was heterogeneous in terms of gender (five males and three females), the second homogeneous (six males) and the last also homogeneous (six females). The group members varied with respect to characteristics such as place of residence, occupation and educational standard. The data collected were analysed using the thematic analysis method within grounded theory.

**Results:**

Five themes emerged as possible explanations for non-compliance: Side effects discouraged patients from taking medication; patients took medication only when they experienced perceived symptoms of hypertension; poor knowledge of the disease and the medication used; lack of support by family members; and difficulty in obtaining antihypertensive medication.

**Conclusion:**

Side effects of the medication, lack of information and support, difficulty in obtaining the medication and the fact that the disease is mainly silent played a major role in the poor adherence to hypertension medication. Sustained health promotion and education should be undertaken at all levels of patient contact to ensure good compliance.

## INTRODUCTION

Hypertension is a serious public health challenge in both economically developing and developed countries.^1^ In 2000, about 26.4% of the world adult population suffered from hypertension and it was estimated that by 2025 the global percentage will have increased to 29.2%.^2^ In Africa, the overall prevalence is about 29.4%, with high rates in urban areas.^3, 4^ In sub-Saharan Africa, hypertension has emerged as a major public health concern in recent years^3, 5^ due to rural-urban migration coupled with modernisation trends, characterised by a sedentary style of life and the consumption of a diet rich in refined carbohydrates and animal fat.^6, 7^

Patients managed at an outpatient rural clinic in Vanga, Bandundu Province, Democratic Republic of Congo (DRC), present with inadequate blood pressure control, with subsequent increases in morbidity, mortality and cost of care. On enquiry, the problem appears to revolve around compliance. No previous studies have been conducted to assess adherence to antihypertensive medication in this setting. This study explored reasons for the non-compliance among patients suffering from hypertension.

## METHOD

The aim of the study was to explore reasons for non-compliance among patients with hypertension. The study was descriptive and qualitative in design, using focus group interviews as a data collection technique.^8, 9^ The setting was the Vanga Hospital, a 450-bed rural hospital in the Bandundu Province, DRC, with 10 doctors and 81 nurses. The hospital receives an average of 16 000 outpatients a year and is the referral hospital for the Vanga Health Zone which serves a population of 209 133 people. Four main ethnic groups are found within the population served, with all speaking one language, namely Kituba. At the time of the study (2006), a total of 400 inpatients were seen on a monthly basis.^10^ The hospital caters for the following patient categories: surgery; obstetrics and gynaecology; paediatrics; internal medicine and psychiatry. The study was conducted among both the outpatients and inpatients.

### Sampling

The study population comprised of all patients suffering from hypertension and who attended Vanga hospital's cardiovascular clinic. The sample comprised of three focus groups consisting of six to eight members each. Purposive sampling was used in the selection of the sample.^11, 12^ The selection was based on the patient's willingness to communicate freely about their medical condition and its management, so as to provide rich information.^13^ Included in the study were hypertensive patients who had been diagnosed six months or more prior to the beginning of the study, who had missed two or more consecutive follow-up appointments for hypertension, and who admitted to being without treatment for hypertension for a period of at least two months. Newly diagnosed patients with hypertension that had been diagnosed less than six months prior to the start of the study were excluded from the study.

### Data collection

Data collection was performed using the focus group interview technique. Interviews were conducted by the researcher and the research assistant, the latter being a professional nurse. All interviews were conducted in Kituba, the local language. Three focus group interviews were conducted from 23 March to 19 July 2006. The demographic details of each participant were recorded, i.e. age, sex, marital status, occupation, residence, date of consultation at the cardiovascular clinic, and the patient's level of education. The first focus group was heterogenous in terms of gender, comprising of five married men and three married women aged between 34 to 71 years. Only two men in the group were employed. The second focus group was homogenous in terms of gender, with six males aged between 51 and 74 years, all married. Three members of the group were employed. The third focus group was homogenous in terms of gender, comprising of six women aged between 38 and 58 years; four married and two widowed. Only one member of the group was employed. The exploratory question in Kituba was: ‘What are your reasons for not taking your medication?’ The discussions were audio-taped and field notes were taken. The audio-taped data was transcribed verbatim, and then translated into French (the official language in the DRC and with which the research team is fully conversant). The data was translated from French into English (the official medium of instruction at the University of Limpopo where the Master's dissertation that initiated this research would be presented). The translation into both languages was performed with the assistance of a specialist in linguistics from the Protestant University of Congo (PUC).

### Analysis

Data collected were analysed using the thematic analysis method within grounded theory^14^. Data analysis was performed in steps. First of all, data were organised in files so that all the materials retracing the interviews were regrouped from the focus group interviews. Data were then reviewed to identify emerging themes, categorising each identified theme according to the content derived from the interview responses. Next, categories identified were re-examined to determine how they were linked (axial coding), as recommended by Corbin and Strauss.^8^ Finally, the conceptual model that emerged formed the story line.

The study received ethical clearance from the Medunsa Research and Ethics Committee (MREC) of the University of Limpopo (Clearance Certificate number: MP 08/2006), and from the hospital authorities of Vanga Hospital (DRC).

## RESULTS

### Themes

Five themes with supporting quotes from the transcripts were identified (Table 1).

**TABLE 1:** Themes and sub-themes

THEMES	SUB-THEMES	SUPPORTING QUOTES
**1. Side effects (discomfort)**	Experience of discomfort	*‘The first time when I took the medication I felt very uncomfortable and I thought that the disease was getting worse’*
	Patient's own decision	*‘When I experienced the discomfort, I stopped the treatment’* *‘When I feel better, I stop taking the medicines so as to avoid the discomfort I experience when taking the medication’*
**2. Subjective feeling of high blood pressure**	Reliance on feelings	*‘When my blood pressure is high, it seems as if I was engaged in some strenuous work and I feel very tired’* *‘It feels as if someone is beating a drum inside my head’. ‘I can't sleep well and my heart is beating fast’* *‘When I feel that the blood pressure is high, I increase the number of pills - I take twice the prescribed dose’* *‘When I feel better, I stop to take medicines, to avoid the discomfort’*
**3. Knowledge**	Medical information not made readily available	*‘The nurse does not tell us about the discomfort when giving us the drugs’* *‘The nurse only told me to return to the hospital if something goes wrong when I take the medicines’*
**4. Support**	Poor family support	*‘I have no person to remind me to take the drug’* *‘I do not receive assistance from anybody’*
	Poor community support	*‘They say that we are witches’* *‘They say I developed hypertension because I killed her sister’ (through witchcraft)*
**5. Access to medication**	Poor staff relations	*‘Sometimes, the person in charge of the dispensary, made it difficult for us to get the medicines’*
	Patients had no time	*‘I am too busy at my work and I do not have time to come to the cardiovascular clinic’*
	Perception about hospital medication	*‘I wish I had more effective drugs than the ones I am receiving now’* *‘The drugs we receive look like sleeping tablets, the real drugs of hypertension are given elsewhere (in private clinics)’*

### Theme 1: Side effects

Side effects discouraged patients from taking medication. The participants expressed in various ways the discomfort they associated with the antihypertensive medication they were taking: *‘The first time I took the medication I felt very uncomfortable and I thought that the disease was getting worse’*. The discomfort experienced tended to override the awareness of the benefits as outlined to them by the health professionals. As a result, they avoided taking the medication as prescribed: *‘I cannot continue taking the pills when I feel uncomfortable’; ‘If I have to work, I'm skipping the morning pill’;* and *‘The drug makes me feel weak’*.

### Theme 2: Subjective feeling of high blood pressure

Medication was taken only when they experienced symptoms of hypertension.

The participants expressed diverse symptoms they believed were due to a rise in their blood pressure: ‘When my blood pressure is high, it seems as if I was engaged in some strenuous work and I feel very tired’; ‘It feels as if someone is beating a drum inside my head’; and ‘I can't sleep well and my heart is beating fast’. This feeling led them to start taking the prescribed medication. Sometimes they tended to overdose themselves: ‘When I feel that the blood pressure is high, I increase the number of pills - I take twice the prescribed dose’. When patients started feeling better again, they stopped the medication: ‘When I feel better, I stop to take medicines, to avoid the discomfort’.

### Theme 3: Knowledge of hypertension

Patients had poor knowledge of the disease and the medication used. The majority of participants had poor knowledge about their condition and their treatment. They said they were not warned about the possible side effects of the antihypertensive medication. They clearly had a lack of knowledge about hypertension and its treatment: *‘The nurse does not tell us about the discomfort when giving us the drugs’*; and *‘The nurse only told me to return to the hospital if something goes wrong when I take the medicines’*.

**FIGURE 1:**
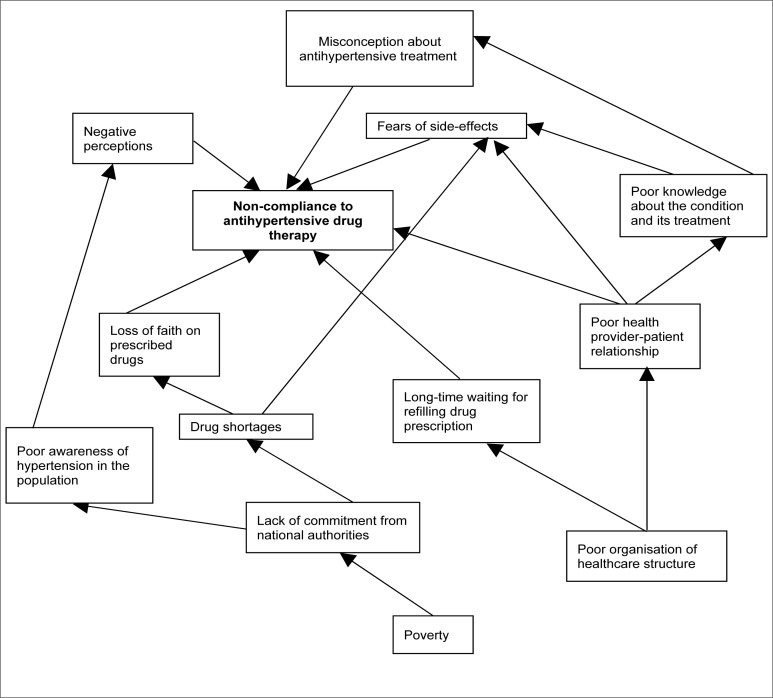
Schematic description of themes

### Theme 4: Support

There was lack of support from family members and the community. Some participants were from families who were not supportive of their hypertensive member: *‘I have no person to remind me to take the drug’*; and *‘I do not receive assistance from anybody’*. There was also the perception from some family members that the hypertensive member brought the condition upon him-/herself by being a bad person: *‘They say that we are witches’*; and *‘They say I developed hypertension because I killed her sister’* (through witchcraft). This viewpoint is shared not only by individual families but also by members of the surrounding community at large.

### Theme 5: Access to medication

Antihypertensive medication was sometimes not available. Patients waited for a long time to receive medication once it had been prescribed. Patients did not have enough time to wait and collect their medication: *‘It takes a lot of time at the dispensary to get the drug, about half a day’;* and *‘I am too busy at my work and I do not have time to come to the cardiovascular clinic’*. The other concern was the manner in which the patients were treated by the staff members at the dispensary: *‘Sometimes, the person in charge of the dispensary made it difficult for us to get the medicines’*. Some patients had misconceptions about the medication being provided at the clinic: *‘The drugs we receive (here) look like sleeping tablets, the real drugs of hypertension are given elsewhere’* (in private clinics).

### Schematic description of themes

Non-compliance among antihypertensive patients has been identified as a complex problem with multifactoral causes – a point raised by several authors.^15^–^20^

In the model presented to integrate the themes identified in this study, the five themes stated above have been identified as contributory to poor patient compliance. Other factors involved are poverty and poor organisational structure. Szirmai, Csaba and Csaba found that improving the doctor-patient relationship is key to ensuring compliance.^21^ Poverty has been identified as an important factor in non-compliance in several studies.^22, 23^ In this setting, the hospital offered a subsidy which enabled hypertensive patients to afford medication. However, when patients needed to acquire medication away from the hospital, affordability became a problem due to poverty.

## DISCUSSION

The study aimed at exploring the reasons for non-compliance among patients with hypertension managed at the cardiovascular clinic at Vanga Hospital in the DRC. The qualitative design enabled the researchers to attain a certain depth of information as the phenomenon under investigation was poorly understood. Crabtree and Miller, as quoted by Reid, ‘have legitimised the qualitative methodology as a powerful tool to answer questions in primary care that have eluded the quantitative approach’.^24^

### Side effects of the medication

The side effects of the medication were most frequently cited as the reason for non-compliance. Svensson and Kjellgren in a qualitative analysis of semi-structured interviews found that the occurrence of side effects featured prominently among the reasons for changing or discontinuing medication among hypertensive patients.^25^ Other authors, although acknowledging that side effects play a role, did not find them to be the main contributor towards non-adherence.^26^ In this study, side effects led to patients feeling worse than they had before commencing the treatment. This led to poor compliance as patients tried to devise their own solution because they had not received prior advice in this regard from the health care workers in the clinic.

### Treatment of high blood pressure

In this study we found that, in response to the subjective feeling of high blood pressure, the participants who had stopped medication resumed taking it again and tended to overdose themselves in an effort to ‘catch up’ on the missed doses. This tendency by patients to change the prescription was also found by Svensson and Kjellgren, who observed that patients tended to adjust or interrupt treatment when experiencing side effects.^25^ In this study, due to the side effects, very few of the respondents were found to be compliant. Salako, Ajose and Lawami investigated the link between the free supply of antihypertensive medication and compliance among patients in Ghana. They found there was no improvement in blood pressure control in spite of the free medication. The subsidy itself did not act as an incentive for satisfactory blood pressure control.^26^ The implication is that other factors that encourage strong compliance to treatment are still to be discovered (including what motivates a patient to adhere to prescribed medication).

### Knowledge

A contributing factor to the misconception of side effects by the respondents in this study was poor knowledge about hypertension and its treatment. Whereas the responsibility for compliance cannot be solely shifted to patients, studies have shown that poor knowledge of patients on their management is associated with low patient education level.^25^–^29^ Low education level leads to poor conceptual understanding of hypertension as a silent killer which manifests in the sufferer when the damage has already been done. In Third World countries poverty is a contributory factor to low levels of education. Poverty also leads to poorly structured or non-existent national programmes on hypertension. In this study, we discovered that at Vanga Cardiovascular Clinic, patient follow-up visits were only intended for the re-issuing of medication, without providing proper health education on the patients’ conditions and the medication they were receiving. This lack of effective health care provider-patient interaction manifested itself in the poor patient compliance to medication. A study by Neutel and Smith demonstrated that, contrary to the case at Vanga Cardiovascular Clinic, patients educated by doctors or nurses tend to be more compliant.^16^

### Support versus non-support

Some respondents stated that they were not receiving support from their families. They experienced a negative attitude from their fellow relatives who regarded the hypertensive person as being responsible for their own condition. Interestingly, witchcraft featured in this regard, where patients were regarded as witches and wizards who were being punished for evil derived from the practice of witchcraft. This is not unusual in African society, as also found by Mabuza, Ogunbanjo and Malete.^30^ Health care workers are therefore challenged to offer scientific explanations to patients regarding their bio-psycho-social conditions.

The respondents also stated that the perception that they were practising witchcraft was shared by the community at large. This belief revealed a lack of knowledge about hypertension within the population. Lack of support from families and the wider community demotivated the hypertensive patients from taking their medication. Szirmai, Csaba and Csaba noted that lack of patient motivation is one of the major factors contributing to non-compliance.^21^

### Drug delivery system

The respondents expressed concern about the long waiting time to receive their medication. Long waiting time has been cited in the literature as a contributing factor to patient non-compliance, and it seems to be one of the explanations for missing appointments.^31^ The clinic operates only one day per week, which restricts healthcare provider-patient contact, and causes long waiting times. The participants also raised their concerns about the medicines available at the hospital dispensary. The list of medications for treating hypertension seemed limited to them. Some patients are aware that there were only a few diuretics and b-blockers. There was no national programme to ensure availability of a wider range of antihypertensive drugs. The participants in this study seemed to respond in a manner similar to that in a study by Gascón, Sánchez-Ortuňo, Llor, et al., where patient compliance suffered because of a shortage of medical supplies.^18^ In this study, the few drugs that were available at the dispensary were viewed with suspicion with respect to their efficacy. Patients felt that the drugs obtained at private clinics were of a higher quality than the ones they were receiving at the hospital clinic.

### Limitations

Since the study was qualitative in design, the findings cannot be generalised to the entire population.^32^ However, these findings can be transferred to other similar settings if a similar methodology is followed.^8^ Failing to keep appointments has been used as a criterion for the identification of non-compliance. This criterion has a high specificity and a low sensitivity, and according to Piñiero et al., leads to the exclusion of respondents with potentially rich information.^33^ The use of the focus group interview technique for data collection has its limitations in the sense that the sample is not representative of the study population as, in this case, only respondents who attended the clinic were used.^34^

According to Bowling, focus group studies have a number of bias types, including the bias of interpretation, interviewer bias, sampling, reporting and language bias as the information was translated between Kituba, French and English, leading to possible loss of information.^35^

### Conclusion

Discomfort from side effects of the medication was the common reason expressed by respondents for non-compliance. The respondents expressed fears of the side effects and in most cases stopped or changed their treatment so as to remain feeling comfortable. Lack of knowledge about the disease and its treatment was responsible for the stoppage and/or alteration of the medication. Some family members and the community expressed a negative attitude towards hypertensive patients, which in turn demotivated the hypertensive patients in terms of compliance. Patients perceived the hospital's range of anti-hypertensive drug treatments as limited, resulting in patients having no confidence in the drugs they were receiving. The poor drug delivery system led to delays in drug supply. Poor health care provider-patient relationships also played a significant role in non-compliance. It is thus recommended that sustained health promotion and education be undertaken at all levels of patient contact to improve compliance.
